# Supplementary material for: The causal relationship between the human gut microbiota and pyogenic arthritis: a Mendelian randomization study
Source: Front Cell Infect Microbiol. 2024 Nov 26;14:1452480. doi: 10.3389/fcimb.2024.1452480 (PMC11629706; doi:10.3389/fcimb.2024.1452480)
Supplement: Supplementary file 1 [file DataSheet1.zip › mendelian test/ebi-a-GCST90027857.csv_leave_one_out.pdf]

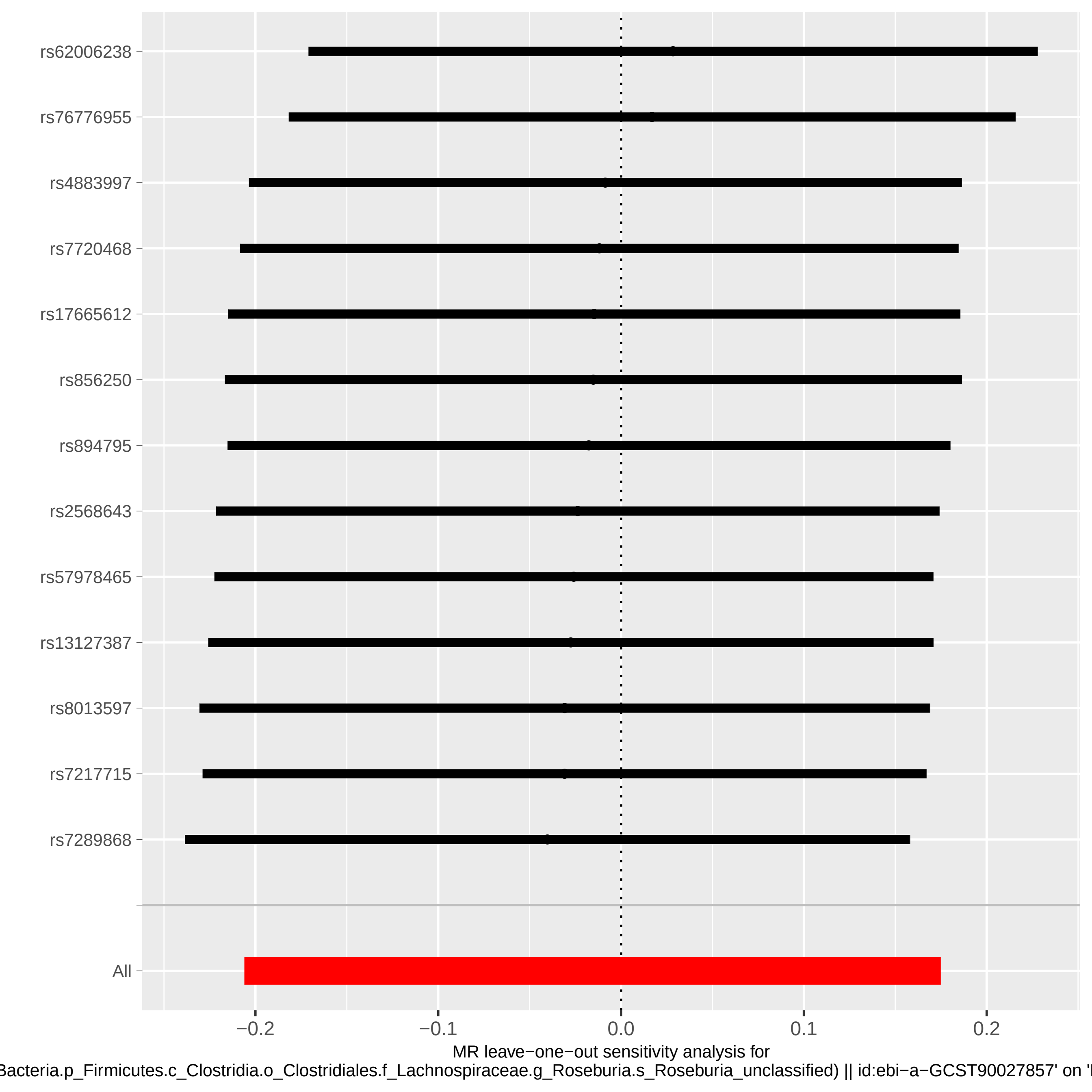

rs62006238
rs76776955
rs4883997
rs7720468
rs17665612
rs856250
rs894795
rs2568643
rs57978465
rs13127387
rs8013597
rs7217715
rs7289868
All
−0.2
−0.1
0.0
0.1
0.2
MR leave−one−out sensitivity analysis for
Bacteria.p_Firmicutes.c_Clostridia.o_Clostridiales.f_Lachnospiraceae.g_Roseburia.s_Roseburia_unclassified) || id:ebi−a−GCST90027857' on '
